# Bottom-up innovation for health management capacity development: a qualitative case study in a South African health district

**DOI:** 10.1186/s12889-021-10546-w

**Published:** 2021-03-24

**Authors:** Marsha Orgill, Bruno Marchal, Maylene Shung-King, Lwazikazi Sikuza, Lucy Gilson

**Affiliations:** 1 Health Policy and Systems Division, School of Public Health and Family Medicine, University of Cape Town, Cape Town, South Africa; 2 Institute of Tropical Medicine Antwerp, Antwerpen, Belgium; 3 Independent, Cape Town, South Africa; 4 London School of Hygiene and Tropical Medicine, London, UK

**Keywords:** District health system, Management, Capacity, Capacity development, Bottom-up innovation, Sensemaking, Sensegiving

## Abstract

**Background:**

As part of health system strengthening in South Africa (2012–2017) a new district health manager, taking a bottom-up approach, developed a suite of innovations to improve the processes of monthly district management team meetings, and the practices of managers and NGO partners attending them. Understanding capacity as a property of the health system rather than only of individuals, the research explored the mechanisms triggered in context to produce outputs, including the initial sensemaking by the district manager, the subsequent sensegiving and sensemaking in the team and how these homegrown innovations interacted with existing social processes and norms within the system.

**Methods:**

We conducted a realist evaluation, adopting the case study design, over a two-year period (2013–2015) in the district of focus. The initial programme theory was developed from 10 senior manager interviews and a literature review. To understand the processes and mechanisms triggered in the local context and identify outputs, we conducted 15 interviews with managers in the management team and seven with non-state actors. These were supplemented by researcher notes based on time spent in the district. Thematic analysis was conducted using the Context-Mechanism-Outcome configuration alongside theoretical constructs.

**Results:**

The new district manager drew on systems thinking, tacit and experiential knowledge to design bottom-up innovations. Capacity was triggered through micro-practices of sensemaking and sensegiving which included using sticks (positional authority, enforcement of policies, over-coding), intentionally providing justifications for change and setting the scene (a new agenda, distributed leadership). These micro-practices in themselves, and by managers engaging with them, triggered a generative process of buy-in and motivation which influenced managers and partners to participate in new practices within a routine meeting.

**Conclusion:**

District managers are well placed to design local capacity development innovations and must draw on systems thinking, tacit and experiential knowledge to enable relevant ‘bottom-up’ capacity development in district health systems. By drawing on soft skills and the policy resources (hardware) of the system they can influence motivation and buy-in to improve management practices. From a systems perspective, we argue that capacity development can be conceived of as part of the daily activity of managing within routine spaces.

**Supplementary Information:**

The online version contains supplementary material available at 10.1186/s12889-021-10546-w.

## Background

Decentralisation debates have a long history in the health sector in low- and middle-income countries (LMICs) [1]. The rationales for decentralising decision-making authority include better coordination of disparate activities, improved use of local knowledge, and strengthening accountability - with the intention to improve the equity, responsiveness, efficiency and quality of health services [2–4]. Over time, the district health system (DHS) has become understood as an important decentralised foundation for a well-functioning and primary health care (PHC)-oriented system [5, 6]. The DHS “*consists of a large variety of interrelated elements that contribute to health in homes, schools, workplaces, and communities, through the health and other related sectors …*. *Its component elements need to be well coordinated by an officer assigned to this function in order to draw together all these elements and institutions into a fully comprehensive range of promotive, preventive, curative and rehabilitative health activities.*” [7].

Many agree that the management and leadership capacity of the lead ‘officer’ (and her/his team) to steward the DHS is a key cross-functional ingredient for strong health systems functioning [7–20]. District managers (DMs) and their district management teams (DMTs) are the middle managers who “*work at the boundaries between senior management and the rest of the workforce*” [21]. They must conduct both ‘sensemaking’ around top-down policies and the changing environment, as well as ‘sensegiving’ to a variety of actors in the district in order to direct change [22]. They are responsible for improving and sustaining organizational performance over time, ‘managing’ the internal activities of the organization and ‘leading’ the staff and external partners in the face of increasingly complex conditions [23]. From a bottom-up perspective, they often shape policy [24].

However, across settings, capacity to manage the DHS is often found to be weak and in need of strengthening [8, 16, 25–28]. The attention paid to strengthening such capacity has resulted in the development of many managerial competency frameworks [29–32] and the delineation of the 12 practices of managers, grouped as (1) leading, (2) managing and (3) governing [13]. Over time, as the DHS has come to be recognised as a complex adaptive system (CAS) [33–35], understanding of the competencies and capabilities that district managers need has further evolved [10, 36–39]. Capacity development efforts have moved beyond the traditional focus on administrative management and health professional practice training. Instead, they have come to consider the leadership skills needed to manage complex systems and intersections with the organisational environment, including both harder (budgeting, planning, monitoring etc.) and softer competencies (communication, trust building, networking etc.) [19, 20, 36, 37, 40–44]. The recognition of the health system as a CAS also demands different ways of managing and measuring capacity development interventions. A systems perspective looks beyond the black box of the intervention, to consider the how and why of capacity development, understanding it as a process of system learning [40, 45]. Baser and Morgan [46], for example, bring a CAS perspective to capacity, moving beyond linear understandings. They define capacity development as an “*emergent combination of individual competencies, collective capabilities, assets and relationships that enables a human system to create value*”.

There are several calls for further research on management and leadership capacity in the DHS. These identify as important: the role and capacity of middle managers in bridging policy and practice; how management practices become part of organisational routines; how capacity development interventions ‘work’ for managers in diverse settings; better knowledge on complex leadership and strategic management of the health workforce; and operational research on how to develop capacity in decentralised systems [8, 16, 20, 37, 47–49].

This paper presents insights from a realist evaluation in one South African district that illuminates how district managers design bottom-up innovations to improve management practices in meetings through simple but profound acts of sensemaking and sensegiving. It provides lessons that can inform thinking on the approaches needed to develop DHS management capacity. Bottom-up policy implementation theory tells us that managers make meaning of top-down reforms based on the conditions in which they work and that they use their own experience, discretion and tacit knowledge to transform policy into practice [11, 50–52]. Making meaning of and interpreting top-down instructions is an act of sensemaking [22, 53]. Sensemaking has to do with the way managers understand, interpret and create sense for themselves based on the information surrounding strategic change [53].

### The local setting: DHS in South Africa

Pre-1994, the health system of South Africa was fragmented along racial and geographic lines. During the apartheid era, deliberate differences in the allocation of funding, infrastructure and human resources between areas and levels of care resulted in inequitable access to health care by the population – and interprovincial and urban-rural inequalities persist today [54]. In 1994, the new African National Congress government faced the massive task of reducing fragmentation – eventually consolidating the health system into one National Department of Health and nine Provincial departments of Health [54]. A new Health Plan for post-apartheid South Africa (1994) laid the basis for the introduction of a district-based PHC system in South Africa [55]. The primary purpose of the new DHS was to involve local people in decision making, to take account of local needs, to overcome inefficiencies in service delivery and to shift from “*administering health services towards improving health and quality of care at the local level”* [56]. South Africa now has 53 health districts spread across its provinces, each led by a district manager who is supported by a district management team [56], comprised of members with different capacities and authorities [15]. Table 1 shows the responsibilities of district management teams in South Africa.

**Table 1:** District Management Team core responsibilities in South Africa

• Identification of client and stakeholder needs	
• Identification of critical health and systemic challenges and understand source of the challenges	
• Take decisions and set priorities (public health interventions)	
• Balance competing demands by taking decisions on key District Actions, which respond to key priorities, client and stakeholder needs and challenges	
• Allocate resources (time from personnel, goods and services and capital costs). Ensure that capacities are matched with planned Actions. Refine the Actions until the allocated resources meet the Actions	
• Monitor and reflect on progress against plans	
• Strengthen processes where necessary (to implement the plan)	

Source: [57]

The DHS in South Africa has achieved successes over the years, but there is still need for improvement in developing the capacity of DMTs to distribute and manage resources [15, 58, 59]. In 2012, the National Department of Health introduced a range of innovations under the banner of ‘National Health Insurance (NHI) piloting’. These innovations focused on strengthening the public health system in preparation for major health financing reforms [60, 61]. Eleven NHI district pilot sites were selected in 2011 on the basis of their underperformance in health outcomes relative to other districts in the country [62]. The innovations proposed/introduced centred on re-engineering the PHC platform and included a call to strengthen the capacity of management in the DHS at all levels [63]. Among the many top-down capacity development initiatives introduced was a hospital revitalisation strategy focused on Hospital CEO capacity. In addition, as identified in our previous research, some district managers used their discretion to develop bottom-up innovations to strengthen management [24, 61, 64].

### Bottom-up innovation

We followed the emergence of such bottom-up innovation for 2 years in two districts to understand the ‘how’ and ‘why’ of capacity from a systems perspective. We present results from one district in this paper. Realist evaluation starts from a programme theory (see Methods). Here, we briefly introduce the key elements of the programme theory, expanding on it in the methods section.

In a district with some of the worst comparative health outcomes in the country which was piloting reforms linked to national health insurance (NHI), a new DM sought to institutionalise functional systems, explicitly focusing capacity development efforts toward improving practices within the routine extended district management monthly meetings. This is a core meeting space for oversight and planning in the district which includes senior managers and invited partners.

### The suite of inter-linked innovation

The DM worked with a combination of existing resources to address challenges within the management team meeting. He designed a suite of bottom-up innovations, understood as “… *the introduction of new elements into a public service – in the form of new knowledge, a new organisation, and/or new management or processual skills. It represents discontinuity with the past.*” [65]. These innovations included: introducing a new meeting agenda that focused on all the health system building blocks; developing job descriptions for former hospital chief executive officers (CEOs) who were sent to work in the district office ‘without a portfolio’; inviting non-governmental organisation (NGO) partners to the meeting to foster shared vision and accountability; enforcement of the Health Management and Information Systems (HMIS) policy to promote information use by managers; and efforts to focus on solutions in meetings not only problems.

## Methods

**Fig. 1:**
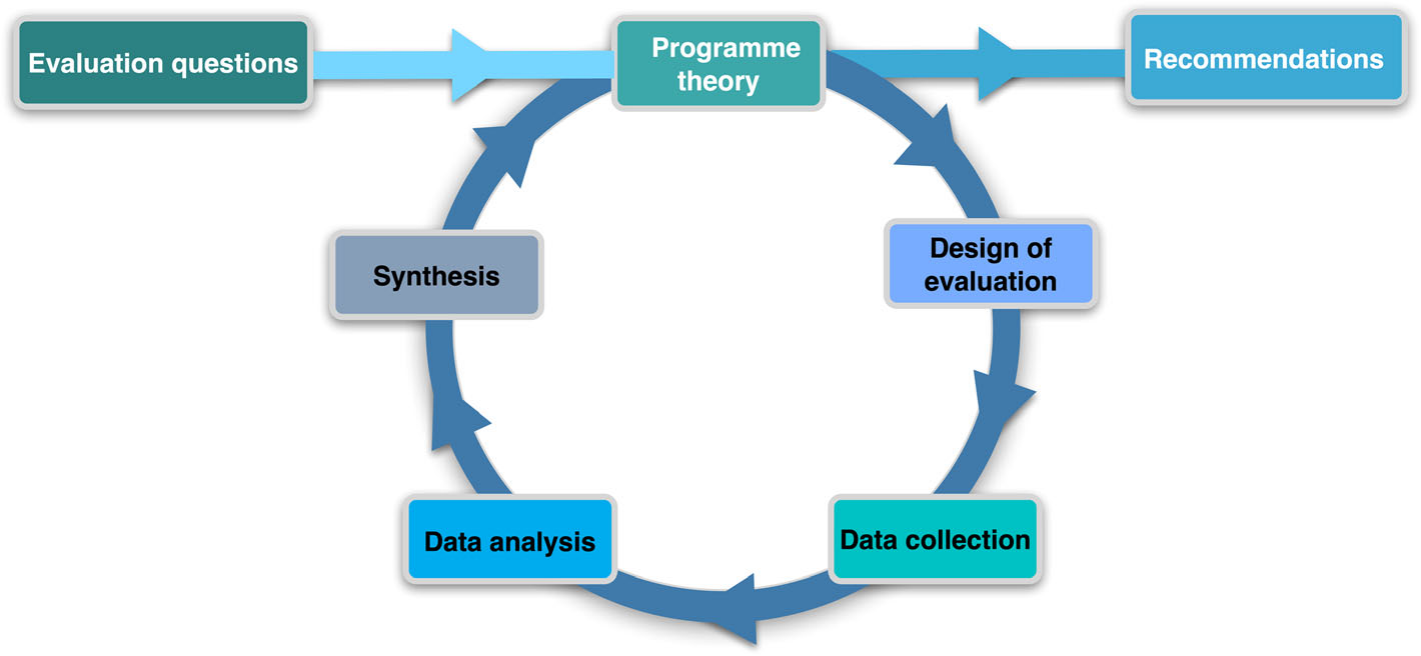
The realist evaluation cycle [66]

We conducted a realist evaluation over a two-year period (2013–2015) in one health district. This study followed the realist evaluation cycle (Fig. 1).

### Study aim

To contribute to an evolving understanding of how to develop management capacity in district health systems.

### Research question

What mechanisms for change are triggered when bottom-up innovations to develop management capacity emerge in the district context and how do these homegrown innovations interact with the existing social processes and norms? What outputs and outcomes emerge?

#### Eliciting the programme theory (PT)

To elicit the PT, we drew on (1) theories of bottom-up innovation and capacity development and (2) exploratory research to elicit the assumptions of key actors who designed the bottom-up innovations in the local context. The full programme theory is presented in Additional file 1.

We have articulated the first part of the programme theory in the background section of the paper, considering theory on bottom-up implementation and a description of the innovation. Here we further elaborate the mechanisms, outputs and outcomes of this programme theory.

#### Hypothesized mechanisms

In times of change, managers, like the new DM, need to challenge the existing ways of working that drive individual and collective action [67]. Managers first engage in sensemaking but must also sensegive to their staff to get them to buy into and enact the innovations in practice, a step that forms part of and precedes an innovation adoption decision. Sensemaking and sensegiving are “*complementary and reciprocal processes*” [53]. “*Sensegiving is concerned with their [managers’] attempts to influence the outcomes, to communicate their thoughts about the change to others, and to gain their support*” [53]. Innovation recipients also work through a series of their own sensemaking cycles before the adoption decision [68]. It is this cog in the wheel of change we seek to explore - the cycles of sensemaking that precede the adoption decision. Rouleau and Balagon [69] identify two strategic discursive competencies of managers. First, ‘performing the conversation’, which includes crafting and diffusing messages in order to influence others, using the right words, the appropriate metaphors and symbols – in ways that speak to the demands and interests of others. Second, ‘setting the scene’, which is about bringing the right people and alliances together; this includes mobilising networks as well as drawing on others for influence and legitimacy, “…. knowing how to set up the arena in which the conversations are to be performed”. Table 2 outlines the micro-practices entailed by these competencies that are “*embedded in tacit knowledge and social contexts*” [53]. These include the notions of carrots, sticks and sermons that are borrowed from political science theory, where they are used to categorise policy instruments for behavioural change [70]. Action, participating in the activity, is another key ingredient for sensemaking [67].

**Table 2:** Four micro practices of strategic sensemaking and sensegiving

Micro practices	
Translating	Translating is an act of authoring, involving selecting the content to be shared and then using material and discursive symbols in the language of the receiver to bring the elements together. Elements and symbols are chosen purposefully to establish shared meaning, managers use their tacit knowledge of people and situations to shape the content.
Over-coding	Inscribing speeches and acts in the appropriate professional and socio-cultural codes of the receiver to reinforce meaning. Different social contexts are home to different social codes, social codes are intrinsic to meaning creation.
Disciplining the client	In routines and conversations, managers produce subjective and emotional effects around the change. Disciplining clients therefore consists of using diverse tactics – including symbolic (e.g. speaking in someone’s language, invoking common cultural roots to create shared meaning), and discursive consciousness (conscious use of implicit knowledge to construct and tell stories – to subjectively influence and convince recipients to adopt change). Through their implicit knowledge managers create sense for others and diffuse meanings around the change. This includes the use of space and body to create an environment which resonates with what is trying to be achieved.
Justifying the client	Providing a set of good reasons for actors to adopt the change.
Sticks, carrots and sermons	Sticks reflect the use of tools to mandate compliance (e.g. regulation). Carrots represent the use of incentives or rewards to motivate for a change in behaviour (e.g. the offer of a subsidy). The use of sermons is the attempt to “*influence people through the transfer of knowledge, the communication of reasoned argument*” (e.g. sharing information).

Source: [53, 70]

We hypothesize that the reciprocal processes of sensemaking and sensegiving will kick-start a generative process of buy-in for new management practices by members of the extended district management team, including senior managers in the district and some NGO partners.

#### Proximal outputs

The ouputs envisioned by the new DM included a well-structured meeting with an agenda that focused on core business; improved use of information by managers for decision making; sharing of solutions by managers; NGO services aligned to the district health plan; and a correct skills mix in the DMT. We only assess outputs in this study. These proximal outputs serve as improvements on the journey to full capacity.

#### Outcomes

The new DM wanted to develop the *capacity* of the management team by improving the practices and processes of the managers in the monthly DMT meeting. We see capacity development as a continuous process, not a time-bound, discrete intervention. *Capacity development* is “*the process of enhancing, improving and unleashing capacity; it is a form of change which focuses on improvements*” (Baser and Morgan, 2008, pg. 3). Capacity as the long term outcome in the programme theory is understood as an “*emergent combination of individual competencies, collective capabilities, assets and relationships that enables a human system to create value*” [46].

While we do not measure long-term outcomes, the Baser and Morgan [46] view on capacity enables us to think about capacity as a phenomena that emerges over time, and that includes a set of interdependent collective capabilities (Table 3) that are needed within complex systems. We anticipate that capacity will emerge over time in the district as a result of the suite of inter-connected innovations.

**Table 3:** Five interdependent collective capabilities that emerge and work together to harness capacity in a system

The core capability to commit and engage	Actors can mobilize resources (financial, human, organizational); create space and autonomy for independent action; motivate unwilling or unresponsive partners; plan, decide, and engage collectively to exercise their other capabilities
The capability to carry out technical, service delivery and logistical tasks	Actors produce acceptable levels of performance; generate substantive outputs and outcomes (e.g., health or education services, employment opportunities, justice, and rule of law); sustain production over time; and add value for their clients, beneficiaries, citizens, etc
The core capability to relate and to attract support	Actors can establish and manage linkages, alliances, and/or partnerships with others to leverage resources and actions; build legitimacy in the eyes of key stakeholders; deal effectively with competition, politics, and power differentials
The capability to adapt and self-renew	Actors can adapt and modify plans and operations based on monitoring of progress and outcomes; proactively anticipate change and new challenges; learn by doing; cope with changing contexts and develop resiliency
The capability to balance diversity and coherence	Actors can develop shared short- and long-term strategies and visions; balance control, flexibility, and consistency; integrate and harmonize plans and actions in complex, multi-actor settings; and cope with cycles of stability and change

Directly from source: [71]

### Study design

We employed a realist evaluation (RE) approach, which is method neutral and allows study designs to be chosen based on their capacity to test the initial programme theory. RE not only assesses outcomes, but explicitly seeks to understand the processes involved in achieving the observed outputs and outcomes [12, 72, 73]. It is the combination of intervention inputs together with mechanisms triggered in context that brings about change. Mechanisms are “*not variable [s] but an account of the behaviour and interrelationships of the processes that are responsible for the change*” [72]. Programmes ‘don’t work’, it is people that make them work [72]. Mechanisms are a combination of resources and reasoning, “*intervention resources are introduced into a context, in a way that enhances a change in reasoning*” [74]. Resources (material, emotional, social, encouragement, etc.) and reasoning alter the behaviour of participants in specific contexts, which then leads to outcomes. As the study design, we adopted the case study design [75] as it allows to study a phenomenon in context as it is being shaped and re-shaped.

This study was approved by the University of Cape Town Human Research Ethics Committee (479/2011 and sub study 746/2015).

### Definition of the case and of the unit of analysis

We adopted a single case study design to “*determine whether the propositions [in our programme theory] are correct or whether some alternative set of explanations might be more relevant*” [75]. The context is the district and the case is defined as ‘the introduction of bottom-up capacity development innovations targeted at the district management team’ to improve processes for managing the district.

### Site selection

We purposively selected a health district to which we had access given the larger project in which this work was nested,^1^ and which had a district management team in place. The new DM was willing to grant us access to himself and his staff and had clear ideas on what he was planning to do to strengthen management.

### Data collection

The first author collected data within the period 2013–2015, monitoring reforms in the district pilot site, keeping researcher notes and capturing key reflections on the district’s context. The process of eliciting the initial programme theory from managers in 2013 contributed to a rich understanding of the context (the interview guide is shown as Additional file 2). We drafted the PT and then member-checked it with the new DM in an additional in-depth interview. To test the PT and to understand the processes and mechanisms underlying the introduction and adoption of the innovations, the first author conducted in-depth interviews with 15 senior managers in the district (all of whom were part of the extended DMT) and 7 major non-state actors (some of whom participated in the extended DMT meetings). The same interview guide was used in the second round of interviews, which focused solely on the bottom-up innovations that had been identified. Based on what we had learned, we developed an additional interview guide for NGO partners (see Additional file 3). Researcher notes on context were used to further interpret findings.

### Data analysis

In realist evaluation, the context-mechanism-outcome (CMO) configuration is used as the main structure for analysis [72, 74]. The transcripts were coded using principles of thematic analysis. Deductive codes included actors, mechanisms (both resources and reasoning, including the micro practices of sensemaking and sensegiving), contexts, processes and emergent outputs and outcomes, as well as elements of the innovation itself [76]. “*People who study sensemaking pay a lot of attention to talk, discourse, and conversation because that is how a great deal of social contact is mediated*” [67]. The process also included looking inductively for any new ideas that emerged in the data.

In the analysis, we moved back and forth between the empirical data and key theoretical concepts. We deepened the analysis by searching for patterns and conjecturing various CMO configurations, moving between the micro-practices within the meeting space and the interaction with the context. Finally, plausible CMO configurations were tested by triangulating a variety of sources of data including researcher notes and observations, and by validation with co-researchers within the project. Interim findings in this paper were presented to some of the senior managers as part of the larger project feedback session in 2016, and the final conclusions of this paper were presented in 2019 to the District Manager who had led the innovation to member-check the analysis.

### Synthesis and comparison of CMO findings with the programme theory

At the end of the analysis phase, we reflected on our findings against the original PT, considering the data, the theoretical literature and the original PT. In this process, we engaged in peer debriefing across the three authors to discuss what we had found beyond the original assumptions captured in the PT.

## Results section

In this section, we describe the general context of the district, the innovations to strengthen management practices, key outputs achieved in the 18-month period November 2013 to April 2015 and finally, we consider the mechanisms triggered in context that generated outputs. The results are summarised in table format in Additional file 4.

### Context and actors

The health district both under-performed relative to the rest of the country in terms of health outcomes and suffered from human and infrastructural under-resourcing as a result of apartheid legacies [77]. The district is considered rural: it is hard to attract staff to work in it and at times there are poor working relationships between the district and the Provincial government.

In November 2013 a new DM with 29 years’ experience in the South African health system (public and private sector) arrived to lead the district. The new DM worked with a *core* district management team (DMT) who met every Monday morning. There was also an *extended* DMT (including the core managers as well as hospital, programme, sub-district managers and other invited guests; in total 24 managers at that time) who met once a month to report, plan and prioritise for the district. There were critical vacancies in the DMT, and three hospital CEOs who had to leave their hospital posts^2^ were sent to work within the district office with no specific portfolio. The DM reflected that stability was needed. Being an NHI pilot district expected to implement several new service delivery reforms made the challenges more complex:

*“I think the preparation for NHI relies heavily on innovation and in order to innovate properly, you need a stable system. This is an extremely unstable system, so you have got to innovate and stabilise at the same time, which I think adds a lot to the complexity of what we do (*The new DM, 09/09/2014).

The extended monthly DMT meeting needed to change, as it was a space mainly used for complaining. The use of information by hospital and sub-district managers for problem diagnosis, decision making, and accountability needed to be improved:

*“I think that there were lots of meetings, or there are lots of meetings that happen, but not lots of structured meetings. Not lots of minutes and not lots of agendas, so you cannot go to a meeting and you sit there the whole day and you don’t have something tangible to show …. We get a lot of whining sessions, but they actually don’t help at all …. That is more the approach than to listen, because you can spend ninety percent of your time listening to or whining, and then only ten percent looking at solutions, whereas we would like to reverse that … It is about looking at the indicators and asking: “Why we are doing well or why we are doing badly? … It has worked before and it is kind of standard practice in functional systems. I am sure it will work”* (The new DM, 19/02/2014).

The information manager (IM) was carrying the burden of information preparation and presentation for the meeting. She had a sense that managers were afraid of working with numbers and this resulted in a general culture of avoidance and deferring queries back to her:

*“… because even things that they can do themselves, they will also say: “No give it to [the] information person.” … They would make it a big deal when it comes to compilation of other reports. Anything that is computer-related, they associate it with anything that relates to numbers. They will just give it to someone to add it in … they don’t want to use numbers”* (Manager 1, 09/09/2013).

The IM already had a huge workload, including managing all the aggregate information, quality-checking data and being responsive to information requests in the district. Additional data capturers had been sent to the district as it was an NHI pilot site, but they did not have the skills to do the work required. In the past, reports had sometimes been generated but the problems managers raised in them were at times not acted upon reducing motivation to produce new reports.

There were also many NGO partners operating in the district, but it was not clear whether they were well aligned with the service delivery priorities in the district health plan (DHP). The new DM felt there was neither a shared vision with all partners, nor an established decision-making platform where decisions could be taken consultatively with stakeholders.

### The suite of inter-linked innovations to develop management capacity

The suite of inter-linked innovations introduced in the extended DMT monthly management meetings to improve capacity were:

- (1a) The introduction of a new agenda that focused on the core functions of the district (‘services’, ‘corporate governance’ and ‘quality’, with time allocated for each item), and the introduction of a routine procedure to support decision making - whereby managers had to produce reports, covering core indicators for reading, which were distributed before the meeting. Additional file 5 presents an overview of key agenda items.
- (1b) An explicit effort to institutionalise the engagement with and application of information by all managers, backed up by the DM’s purposeful enforcement of the national District Health Management and Information Systems (DHMIS) policy. Linked to this, the DM also established the routine procedure that managers must first investigate problems by collecting information on the ground before bringing them to the monthly meeting, and be ready to discuss solutions and progress (or lack thereof).
- (1c) The routine procedure that NGO partners in the district would attend the extended district management meeting in order to support coordination and accountability, as well as discuss their activities directly with the DM.
- (1d) Defining job descriptions for the ex-hospital CEOs newly posted to the district office describing their purpose in the team; as well as attempts to fill critical management vacancies in the team.

### Outputs

By 2015, 18 months after the new DM’s appointment, senior managers and district partners who attended the monthly meetings confirmed that the innovations had resulted in an emerging set of improved management practices.

#### Output 1a

A new extended DMT agenda with a structured format was being routinely applied, managers had to present on core system issues and meetings were being time-managed.

*"Yes, we present but we are being given a chance, we are being informed earlier on that you are expected to present in such-and-such a DMT because of the time schedule and there are a lot of them here. So, it doesn’t become possible for us all to report. For instance, there’s a lot of, the NHLS, there’s pharmaceutical, there’s the information officer who gives a summary report for the activities that happened in the districts. Then we input or respond; when you haven’t done well, you indicate what causes the deviations from targets and how are you going to improve on those things. And if we don’t present the actual status ourselves, it appears".* (Manager 7, 02/10/ 2015).

Already by the end of 2014, at least 15 managers were preparing and submitting reports to the DM, who then decided both what would be discussed in the meeting and which reports would be circulated in preparation.

*“so what we are trying to do now is have a structured agenda, not a reactive agenda, a structured agenda where you have reports that you prepare and then the line management people that attend have to interact with those reports*” (The new DM, 19/02/2014).

The hospital managers and sub-district managers as line managers were expected to read the reports to empower themselves. Nonetheless, getting managers to engage with information in the reports was not easy. The DM identified two challenges: he was not fully satisfied with the make-up of the reports and not all managers had read the reports as needed before coming to meetings;

“*because progressively we are going to start making decisions based on that and if they don’t read those reports* … …. *we are now at the point where we are kind of saying read your emails, read your reports etcetera*” (The new DM, 19/09/2014)

#### Output 1b

The application of information for decision making was now part of managers’ performance contracts as per the Health Management and Information Systems (HMIS) Policy. There was an improved use of information to diagnose problems, monitor progress and support forward planning in the extended DMT meetings by sub-district managers. The IM and another manager in the DMT confirmed that, in 2015, service delivery information was being presented and discussed in the meeting and that managers had to account for targets. This process remained in place after our final evaluation period.

“*We continued with what [the new DM] has started. We look into the indicators and the performance of the district, the subdistrict and the hospital CEOs, they do make some presentations so that we are able to identify gaps and formalise some strategies to work around the gaps - we’re still continuing.*” (Manager 6, 17/05/ 2015).

While problems were still brought to meetings, there was a proactive effort to identify solutions in the meeting:

*"So now, at least people, even though not everybody, but some are able to say, okay, we have got a challenge of transport – how about if management could talk with [the] municipality so that we can join vehicles together when they are going to ward A, maybe we got to ward A, all of them. Starting from that integrated planning there."* (Manager 8, 24/03/2015)*.*

*"I have to get assistance from the people who are actually doing the immunisations, what was the problem? Were there vaccines that were not available, for instance; or was there something that made them not be able to come to the facility?"* (Manager 7, 02/10/2015).

#### Output 1c

Improved capability to relate and partner with othersStaff of the large NGOs in the district met with the new DM personally to report on their district activities, and subsequently, a growing number of NGOs were reported to attend extended DMT meetings to present and discuss their progress. However, we primarily observed interactions with representatives of two large NGOs that had been present in the district for more than 5 years, had their own funding and were actively implementing health programmes and/or were directly engaged with senior management in the district toward health system strengthening. These NGOs also participated in developing the DHP to ensure shared planning and vision.

“*Yes, I was part of that stakeholders [mapping] meeting and we all [NGO partners] presented the work that we are doing, the challenges and the successes that we have had. And on a monthly basis we used to give him our progress reports in the DMT meetings*” (NGO partner 1, 18/05/2015).

" *…. they [NGOs] are actually invited to make inputs [into the DHP] and also to look at the priorities of the district when they are going to be doing that. So their plans must actually be part of what the district plan is*" (Manager 4, 1/10/2015).

Formal invitations to partners had also become routinised.

"*Ja [yes], I think mainly it’s [NGO partner 1 & 2] who are attending those district management meetings, though it’s continuously growing in terms of who is attending those meetings.*" (Manager 4, 1/10/2015).

An NGO partner who had been part of the DMT meetings before 2013 (when the new DM arrived) noted that as partners had to present on their activities when attending the meetings, accountability amongst NGOs improved (NGO partner 2a, 2/10/2015).

#### Output 1d

The new DM filled at least two key senior management posts that had been critical vacancies: an HIV/AIDS, STI and TB (HAST) manager and a quality assurance manager. Also, the hospital CEOs who had been redirected to the district office were given clear job roles^3^ linked to their competencies and the needs of the DMT.

### Mechanisms for change

#### Initial sensemaking by the DM

The arrival and initial sensemaking by the DM were both a trigger and a mechanism in improving management practices in the DMT meeting.

*“Look, when I first got here, we went through quite a long process of saying: “What is the ideal organogram that is needed at district level? What are the ideal processes needed at district level to ensure that we are able to have a strong management team that can take us into the NHI?” Therefore, I think it does depend a lot on what people we’ve got. I think there needs to be a standardisation of processes, because the way I am doing things, it is pretty similar to the way they do it in the [previous Province he worked in], but chatting to my colleagues from other provinces, it is not the same and I think there needs to be a standardisation of the management processes. There should be some space in between for us to express our individuality and so on, but essentially there needs to be an improvement in the standardisation”* (The new DM, 09/09/2013).

The new DM drew on his personal resources, including tacit knowledge and experience in the public and private health system in another province to design the suite of inter-connected innovations. He did not believe that more resources would by themselves improve district performance and instead judged that inefficiencies in the public sector could be dealt with through system improvements. The new DM explained where the idea for the structured agenda came from:

*“My little thing to keep me focused, there is a thing called the district management accountability framework, which over the …. five years, … that I was a manager in [Province X], we progressively developed a series of things that need to be in place for a health system to be functional. So, we documented you know, the governance, management, leadership … as I was saying, those things are the pillars of … what is it … [the] WHO building blocks, but having lived through the … development of it, I understand it in a particular way. It is ... management, governance, leadership, it is service delivery, it is critical support functions, and it is quality. Now … and below that, I can see the headings … and that is the agenda for the DMT* (The new DM, 09/09/2013).

*“So, I think the vision comes from … a lot of the vision comes from what I have seen in reality in [Province X]. A lot of the vision [also] comes from what I have seen in reality in the private sector”* (The new DM, 9/09/2014).

#### Introducing a new agenda in the extended DMT meeting: sensemaking and sensegiving as reciprocal processes

The DM ‘disciplined’ the DMT meeting space as part of sensegiving to others – as shown in Table 2, “*discipline comes from a meticulous organisation of gestures, words and objects that permits optimal use of space, bodies, and thought*” [53]. He employed tacit and experiential knowledge of meetings and agendas to structure proceedings in the space, the information managers summarised the comparative data and time was allocated for managers to speak to their performance, reinforcing accountability.

The DM translated and framed the need for a new agenda by drawing on familiar organisational-cultural codes of the health system, including discourses such as ‘core business of health’, ‘patient care related’, ‘indicators’ and ‘PHC’ and ‘performance’ – which can be seen as the careful crafting of ‘normative sermons’.

"*You know, when he came, there was much more focus around the core business in meetings, than to simply discuss how much money we have spent around HR, around that, and so on. Remember, we are having this business of being the Department of Health, so everything must be patient care-related. Now once you talk the performance indicators, you talk PHC, hospital indicators, that’s fundamental – because we can say our department is existing not because of various other things but because of the performance. I would say in relation to that I'm still very much pleased "* (Manager 2, 25/03/2015).

For one manager, working closely alongside the DM (proximity to change) enabled an understanding of the need for change:

*"Maybe one will be saying because I was really always close to this office and having that advantage of knowing why there is this initiative, why we should change – I would say starting from you say the nature of our agenda items in the DMT.”* (Manager 2, 25/03/2015).

The DM over-coded, drawing on familiar organisational socio-cultural codes as a ‘stick’, noting that the ‘auditor general’ (a powerful figure in the bureaucracy) can *check up* on the use of information and the focus on performance in meetings by looking at the agenda, effectively using hardware of the system as a stick linked to accountability.

"T*he DMT meetings might have been held every month, but if in the minutes and the agenda, there’s no … .. agenda items around the information or data management, then you cannot say you are discussing your performance – because it’s not showing in the agenda and minutes. So, that’s what [the new DM] emphasised all the time.*" (Manager 1, 09/09/2013).

The new approach to meetings encouraged active participation by senior managers, whilst simultaneously facilitating their buy-in to the new practices through the process of ‘doing’. Managers appreciated that they were no longer tired in meetings because of long drawn-out processes. Increased participation provided more ingredients for sensemaking and sensegiving, which triggered the motivation and self-efficacy of managers.

*"Yes, because before the subdistrict managers were presenting, the CEOs were presenting – so when the last one is presenting, you are no more listening. It’s already four o’clock, so you are tired. So the way he did it – it’s for the information manager to present comparing the subdistricts, not for subdistricts, for [sub-district A] to present, then one for sub-district B to present, because at the end, you won’t be able to see how do they work comparing them, and where to give assistance. The way he did it is for the information manager to present and show us which subdistricts doesn’t perform well in what. That has really helped us. Like they are also doing it today in preparation of the DMT on Thursday."* (Manager 8, 24/03/2015)*.*

These actions were complemented by the preparation and pre-reading of reports, which reinforced the use of information and, together with the requirement to present problems with potential solutions, fed into a more structured agenda.

#### Embedding the use of information for problem diagnosis and problem solving: sensegiving and sensemaking as a social process

The DM used his positional authority and employed over-coding, drawing on the professional codes of the bureaucracy [public policy], to create shared meaning around information use for decision making. The National Health Management and Information Systems policy (policy hardware) also served as a ‘stick’. The new DM enforced it to justify why managers must use information and monitor performance in their daily practice. Information use also formed part of their performance contracts as per the policy. He matched this with a sermon approach, taking the time to visit managers at facilities, together with the Information Manager (IM). The latter had institutional memory given her working history in the district and was familiar with policies and staff; she also reinforced that they ‘must’ comply with government policy.

"*They [the managers] were fine because we were also emphasising to them that it’s not any person’s choice, because it’s a policy issue which, though we were trained on it, but in terms of implementation, you were not implementing it as expected. But now, that [was coming from] from the district manager" when [the new DM] went around.”* (Manager 1, 09/09/2013)

Including the IM in his visits was symbolic in ‘setting the scene’ as the IM legitimated discussions, was always highly motivated for change (despite not having had the authority to enforce improved information practices) and knew the content of the HMIS in detail. To improve information use and accountability in the DMT meeting, the new DM drew on his positional authority and introduced a requirement that the sub-district manager ‘sign-off’ data from the facilities before sending it to the district office.

*“They* [sub-district managers] *are more responsive, especially when it comes to the variances that we are showing them, because they are the only people that should tell us the reason as to why it is like this.”* (Manager 1, 09/09/2013).

The planning manager, identified as exceptional by the DM, was tasked with reviewing all the data from facilities to identify any obvious discrepancies. The DM then employed ‘sticks’ to reinforce the importance of data by writing letters to each facility manager or sub-district manager, saying either 1) your data was late, 2) your data was not complete, 3) your data is not believable in the following areas (…).

However, the information management changes had not yet impacted at the facility level, the planning manager has picked up similar challenges again:

“*So, she is now … she has given me the second month’s letter, and it is almost identical to the first month’s letter.*” (The new DM, 19/09/2014).

Sensemaking and sensegiving for information use was also reinforced by the working environment. Some managers had been permanently appointed to their positions during the tenure of the new DM. The IM felt ‘being permanent’ supported responsiveness and accountability in the meeting, as when managers are in ‘acting’ positions, it is easier for them to say they are ‘only acting’, avoiding accountability.

Since NHI piloting began, additional resources for performance monitoring were introduced by the National government and the Provincial government, including templates for monitoring and evaluation and sets of preparatory activities for meetings. All managers in the DMT had been given computers and 3G data cards. The IM was hopeful that the new technology would enable better practices by the managers. She felt she needed to be released from the dependence of managers on her for information:

*“Yes, because in those pivot tables [shown on the computers], all the indicators for various programmes, they are there. So the managers even [can] now compare quarters to look at the performance of sub-district A versus [B] sub-district … to see areas that are alarming and as well as for them to be able to act up on the data that they see and it’s also assisting me as information manager, even if I am not there.* (Manager 1, 09/09/2013).

However, there were still challenges to using information for decision making in the DMT, including some managers’ lack of trust in the data. The DM tried to address these reservations by using an example of a project where data had successfully been collected and verified to illustrate that it is possible to change practice and get good data.

He also used his positional authority (system hardware) to encourage managers to present proposed solutions, based on insights from the ‘ground’, in the meeting,

*"Ja, people were focussing on challenges. Really their focus was specific to challenges. Like they are doing now, [they] don’t have vehicles to reach area 1, so at the end what he was saying is “when you have got a challenge, come up with a proposed solution”. It mustn’t be just a challenge being thrown because you need to think what is it that can help you to change."* (Manager 8, 24/03/2015)*.*

However, it was not always easy to make people focus on solutions. Doctors’ accommodation was one intractable problem that seemingly had no solution:

“*So, people started getting a little bit edgy. They said “what is the point of telling this guy that we have got a problem, because he actually can’t do anything about it”, you know and it is that kind of a … situation*” (new DM, 19/09/2014)

When a problem was resolved, the team were asked to share lessons in order to generate collective learning and thus contribute to the collective capabilities of the team.

#### Sensegiving to NGOS: crafting and managing key relationships to attract resources and support

In 2013, the new DM used his positional authority to host a stakeholder meeting for NGOs to present to him what they were doing in the district, what progress they were making and to remind them of their role as supporters in the district. They were told they would be invited to the extended DMT monthly meetings to present on their work to ensure objectives and progress would be aligned to district goals – effectively reinforcing ‘the disciplining of the space’. His actions were supported by many managers in the DMT:

*“They [NGOS] don’t have priorities; it’s the district that has priorities – they are here to support the district to achieve the set targets on those specific priorities.”* (Manager 5, 1/10/2015).

For the NGOs who supported these actions, the new DM tapped into shared meanings and, in some cases, a history of working relationships (for example, generated by sharing office space with the NGO). They felt he was working hard at working together and that he gave them a voice in these processes. He, thus, also tapped into their intrinsic motivation. In this research we interviewed four staff members from two supportive NGOs:

*“Everybody had a voice. Everybody had a voice, all the partners had a voice. We felt part of the plan, and so we were prepared or we managed to own the plan."* (NGO partner 1, 18/05/2015).

*"As a partner we have to compromise. ( … ) As a partner we have to be flexible all the time, because we are here to respond to the needs of the DoH. So, if you are not doing that, then the relationship between yourself and the DoH might turn a little bit sour; so you have to ensure that you’re flexible all the time."* (NGO partner 1, 18/05/2015).

“*No, he was not a difficult person because he had the best interests of the department at heart*” (NGO Partner 2b, 18/06/2015).

The DM told NGO partners who did not want to create a shared vision that he would report directly to their funders, using sensegiving ‘sticks’ to influence participation.

“*We are actually more explicit to them, and said “if you don’t talk to us, then we write to your funder, saying that you are not helping us, then they can send the money somewhere else”, because everybody comes and they think the answer is training.*” (The new DM, 19/09/2014).

Some managers were wary of including NGOs in DMT meetings, given negative experiences of media reporting prompted by NGOs. However, the new DM successfully justified the need for inclusion using his experiential knowledge:

“*Really, it started working. He invited partners, even the partner that we didn’t like a lot, Partner XXXX. So, we felt that these are the people that normally write negatively about the department of health – then why are they here now? But the way he explained it ... because they were part of the meeting and they know what is happening, they have inputted in relation into what is supposed to be changed. … It really worked; I think it really worked, because otherwise we didn’t like the idea, but we saw that as fruitful.*” (Manager 8, 23/05/2015).

As part of his plan, the new DM originally requested one of the large NGO partners to steward all the NGOs in the district as “*they must be guided as to what the needs of the district are*” (The new DM, 19/09/2014). But as this approach did not work because not all NGOs were pulling in the same direction, he then drew on his planning manager, who had a long history working in the district and long-standing relationships, to take coordination forward. The DM thus employed distributed leadership toward the overall goal.

*“[The planning manager] ensures that we plan with our partners; we do reviews with our partners."* (Manager 6, 17/05/2015).

However, the district NGO coordinator felt somewhat left out of these new processes, as he was not a senior manager and did not attend extended DMT meetings. He also primarily coordinated Community Based Organisation Organisations (CBOs), rather than large NGOs - smaller organisations receiving subsidies from the Provincial government and monitored by the district office.

The DM also used familiar professional codes and discourse to encourage NGOs to understand that they had to participate in the development of the District Health Plan. This helped to create shared meaning about the importance of shared vision and accountability in the district:

"*Firstly, [the new DM] told us that what he needs is a consolidated plan for the DoH and for the partners as well. As partners, we have our own operational plans that talk to the objectives and the targets that have been set up by our funders, and there are certain indicators that we need to focus on. Same applies to the DoH, because they have got some indicators that they need to focus on. So, [the new DM] said “with all your plans that you have, they need to be integrated into our master-plan so that we can have one plan that we are going to support and implement as district [X]. So we found that very valuable because with all the plans that we had, we had an opportunity to express our concerns and maybe the needs that we might have as partners for the kind of support that we are expecting from the DoH.*" (NGO partner 1, 18/05/2015).

Other mechanisms in context that facilitated sensemaking and sensegiving included the ongoing work of a large NGO specifically placed in the district to provide technical support to the district as an NHI pilot site. Some donor-funded projects also intentionally and actively sought to build working relationships between themselves and members of the management team (e.g. a UNICEF project).

An NGO partner noted that strong partnerships are built on good relationships:

“*Make good relationships with people, be flexible and try and understand the other’s opinions. Don’t be a know it all - acknowledge we learn from them and then learn from us. Be yourself and present yourself as you are.*” (NGO Partner 2b, 18/06/2015).

Despite the improvements experienced, persistent ongoing challenges for partner NGOs in the district included their limited power to hold staff in the sub-districts they supported accountable, where, for example, staff showed lack of urgency.

#### The number and distribution of managers in the team: negotiation as sensegiving

To fill a critical vacancy (in this case a quality assurance manager) in the DMT, the DM used his positional authority and negotiated within his resource envelope rather than pushing the Provincial government for more money:

“*I have weighed up the benefit of one post above the other one, and said I am giving you [the Provincial government] the money for a quality assurance manager, … I have got a TB manager that resigned, and I said TB and HIV should actually be under the same deputy director. So, I am taking that TB money and that is quality assurance money.*” (The new DM, 19/09/2014).

This approach of prioritising among management posts was contested as some senior managers felt that posts at the same level simply could not be ranked (e.g. occupational health and safety against an HIV manager). But, using his positional authority, the new DM asked managers to rank their posts from 1 to 10 in order to create shared meaning. Using his implicit knowledge, he tried to create sense for others and diffuse meanings around the change – to influence and convince recipients to adopt change. Whilst acknowledging the reluctance of managers to do the ranking and his own discomfort in ranking posts he believed were all important, he noted that it had to be done given budget shortages.

“But you … as a leader and manager, you have to make tough decisions” (The new DM, 19/09/2014).

The DM noted some said he did not push the Province hard enough for more resources, but he drew on his knowledge resources to arrive at a decision:

“*I come from a different school of thought, but I mean to be fair, there are people that say I don’t argue enough for more resources and that is based on … I attended a course on efficiency and so on and he [the lecturer] said the worst thing that you can do for a dysfunctional system is to throw money into it … It makes it more dysfunctional. So, I have been … when Province says I am not giving you money, I say okay.*” (The new DM, 19/09/2014).

For the Hospital CEOs deployed to the DMT without portfolio, the DM considered their skill set and then wrote each 1 a role description for a portfolio of work where they could use their skills, purposefully enhancing the collective capabilities of managers within the DMT in the process. In this, he drew on a common cultural code in the workplace of having a ‘job description’ to facilitate a sense of collective purpose.

*"He couldn’t get formal job descriptions because job descriptions come from the provincial office ..[but] … he looked at those who were additional to the establishment and then from there, he managed to allocate them in areas where he was seeing that there are gaps … So, from there, you will be able now to come with what you are supposed to be doing.*" (Manager 8, 24/03/2015).

#### Did practices continue over time?

The DM who designed the innovations left the district in late 2014, but his successor as DM continued with the innovations in 2015. This new DM reflected that they drew on the courage instilled by the previous DM when applying for the leadership position, as well as recent leadership training. We asked if the changes in the use of information introduced by the previous DM was making things better:

*Yes, it does because that’s what we are continuing even with …. , we continued with what [the previous DM] has started. We look into the indicators and the performance of the district, the subdistrict and the hospital CEOs, they do make some presentations so that we are able to identify gaps and formalise some strategies to work around the gaps - we’re still continuing.* (Manager 6, 17/05/2015).

In 2015, the new DM also confirmed that the Planning Manager continued to ensure that planning and review processes continued with partners. Similarly, the Hospital CEOs deployed to the DMT without portfolio continued to work within clear role descriptions to ensure they functioned as an effective part of the team:

*"Then I am able to allocate them to those areas. So, they’re kind of busy there, because once you don’t utilise one, he becomes demotivated and feels as if he’s worthless. But now, we are utilising them fully."* (Manager 6, 17/05/2015).

Nonetheless, the new DM was not naïve about the broader contextual challenges faced in leading the district in 2015, including key leadership vacancies in the hospitals (and in the district more broadly) and challenges related to clinical governance in some hospitals:

"*So, there are those kind of weaknesses that affect the progress and stability in the district*" (Manager 6, 17/05/2015).

"*So, another weakness is you see there’s a lot of staff turnover in the whole district, especially clinical people, professionals, the nurses. Because you will appoint a hospital manager; while you’re appointing this one, the other one says I'm resigning, I'm going. So it’s those kind of things that are threats now – I've done the weakness, the threats*" (Manager 6, 17/05/2015).

More positively, the new DM mentioned that a new key NHI liaison official had been appointed at the Provincial government, which helped them stay on top of NHI processes in the district.

## Discussion

Amidst challenging contextual conditions and the implementation of top-down NHI piloting, this paper illustrates how a new district manager drew on systems thinking together with tacit and experiential knowledge to design bottom-up innovations to improve management capacity in monthly management meetings. The innovations, together with the agency of the DM, triggered simple but profound micro-practices of sensegiving and social sensemaking among other DMT members. In turn, these triggered a further, generative process of buy-in and motivation among managers and partners to engage in improved management practices in their monthly meeting, unleashing and harnessing capacity in this routine structure (the meeting).

The research thus highlights (1) the individual competency for systems thinking needed by those in sub-national management positions, who must develop capacity bottom-up to manage district functioning; (2) the mechanisms of sensegiving and social sensemaking that trigger motivation and buy-in of district-level managers and NGO partners and (3) bottom-up capacity development as an emergent process in the daily routines of the DHS. These points are discussed further below.

### The competency for complex sensemaking

The DM’s competency for sensemaking in context was a key factor underlying the design of the bottom-up innovation in the experience reported here. Sensemaking has to do with the way managers understand, interpret and create sense for themselves [11, 53, 69]. Managers may adopt a linear or a complex frame to drive sensemaking and interpretation in context [40]. The DM applied systems thinking, for instance, when targeting a routine structure (the meeting) that brought managers and partners together to work across health system functions and silos to manage the district collectively. Systems thinking is an approach to problem solving that views problems as part of a wider dynamic system, “*demanding a deeper understanding of the linkages, relationships, interactions and behaviours among the elements that characterise the entire system*” [78]. Other experiences of capacity development offer insights on how to build capacity for systems thinking. In Ghana, a study of a programme to develop leadership capacity found that teaching systems thinking only as a tool rather than embracing it as an embedded practice failed to develop the new mental models needed [79]. System leaders need to develop and apply three key capabilities: “*their understanding of the system that shapes the challenge they seek to address; their ability to catalyse and support collective action among relevant stakeholders; and their ability to listen, learn and lead through coordination with and empowerment of others*” [80]. The systems thinking competency demonstrated by the DM was informed and complemented by his formal training, as well as his tacit and experiential knowledge of the health system. Together, these individual competencies allowed the DM to design a suite of bottom-up inter-connected innovations to build the capacity of the extended DMT. The DM’s individual competencies thus also contributed to the growing capacity of the DMT.

While we did not measure long-term outcomes, we argue that the outputs observed in this case study are likely to contribute to building capacity in the management team over the long-term, defined as an “*emergent combination of individual competencies, collective capabilities, assets and relationships that enables a human system to create value*” [46]. In their research, Baser and Morgan [46] found that capacity *emerges* out of multiple relationship and that capacity has both technical, organisational and social aspects – which cannot be addressed through exclusively functional interventions. They note that some are sceptical of taking a system approach to capacity development given that the operational implications can be challenging. However, the operational guidance they offer those supporting capacity development includes: (1) given that the future is largely unknowable in complex systems, settle for ‘good enough’ and allow for exploration in the early stages of capacity development; and (2) as “capacity cannot be assembled like a machine”, focus on emergence and opportunities and promote self-organisation for capacity [46].

### Sensegiving and social sensemaking

Recognising the DHS as a CAS informed our approach to investigating capacity development, and required us to look at the software of the system (knowledge, relationships, norms, communication), the intersection with hardware (positional authority, public policy documents) and how together they serve as sensegiving tools that drive an ongoing process of capacity development [9, 46, 81]. *“Sensegiving is concerned with ... [managers’] attempts to influence the outcomes, to communicate … thoughts about the change to others, and to gain their support*” [53]. The micro practice of sensegiving included the use of sticks, such as the DM drawing on positional authority to shape accountability in the meeting, and enforcement of the HMIS policy, and over-coding using discursive symbols, such as ‘the auditor general’. To trigger the motivation of managers and partners, the DM also employed sermons, created shared meaning by taking time to justify and translate the need for new management practices, including visiting managers in their workplace, gave voice to partners in meetings and employed relevant discursive symbols (performance, core business). He also disciplined the space by using an agenda to systematise the processes in the meeting and further drew on distributed leadership to create an environment that reinforced the overall goal [53]. As these experiences demonstrate, complexity-sensitive managers adopt a contingency approach to leadership, balancing transactional, transformational and distributed leadership styles based on the needs of the situation and problem, adapting leadership practices to fit context [37]. A study on the daily management practices of sub-district managers in South Africa found, for example, that improving practices in daily routines, such as facilitation styles in meetings, minute taking, etc. required a set a software skills to nurture and engender “*relationships of constructive accountability … that support persistent and adaptive problem solving aimed at enhancing service delivery and patient care*” [41]. We posit, then, that software skills are critical aspects to be considered when designing and evaluating capacity development innovations.

Sensegiving was strengthened by the DMT members’ proximity to change (working alongside the DM), as well as by their engagement with new practices. In other words, ‘doing’ triggered appreciation for the new practices leading to motivation and renewed self-efficacy in managers and partners - and it triggered a generative process of buy-in. Actions also provide raw ingredients for sensemaking by generating stimuli or cues … “*action serves as fodder for new sensemaking while providing feedback on the sense that was already made*” [67]. Sensegiving and sensemaking are “*complementary and reciprocal processes*” - staff will go through a series of cycles of sensemaking before making a decision to adopt an innovation [53, 68]. Sensemaking is not only an individual act, it is also a social process that is ongoing and recurrent in organisations and that is influenced by contextual factors [67]. In our study, the introduction of NHI piloting came with additional technology to support information use, training on information use in facilities and the placement of the large NGO in the district to provide technical support. These other efforts to build capacity in the context complemented the new DM’s suite of inter-connected innovations.

The outputs of these processes are the emergent practices and processes within a routine meeting structure that reflect improved DMT capacity.

### Proximal outputs and emergent capacity development

Taking a CAS or systems perspective on capacity development in this research has enabled us to look beyond the “input – blackbox - output” model of capacity development [46]. It has allowed us to identify how a space between the health system building blocks/functions, the monthly management meeting, itself shaped by history and context, emerged as a site of innovation and anchor for capacity development within the DHS.

While there are growing efforts to understand how to develop district/health management capacity through CD interventions and courses from outside districts [79, 82, 83], we posit that from a bottom-up perspective, capacity development can be seen as an everyday act of managing. This may refocus attention to the challenging role of daily managing critical vacancies, developing support systems, holding well-functioning meetings for better planning, establishing clear role descriptions and knowledge of one’s role in a DMT [84, 85]. We argue that bottom-up capacity development initiatives anchored in daily routines have the potential to circumvent some of the challenges identified in external, top-down CD initiatives. These include finding time in busy schedules to attend training, additional resources needed to convene new activities, the duplication of existing structures and/or processes in a district and the limited understanding of capacity development as a bounded project that is finished when the project is over or the convenors leave [49, 79]. Homegrown CD activities allow for longer time frames and can potentially deepen local actor ownership and voluntary commitment to CD strategies, both of which are necessary for sustained capacity [71]. We acknowledge that this type of workplace-based capacity development can work in combination with other, and external, forms of training and learning, such as classroom-based learning or e-learning combining theory and practice [86–90]. We also note that some of the general challenges facing innovation in the public service include risk-averse attitudes, coordination problems, opposition to innovation in general and the doubts of stakeholders. To deal with the unexpected challenges likely to arise in implementation, slack should be built into the innovation process [65, 91].

Gilson et al. [92] present a rare empirical example of bottom-up capacity development. They worked with local managers to implement and co-create an intervention to improve governance at the sub-district level. The intervention focused in part on the management of meetings and decision making and included rotating the meeting chair, positive rounds and managing time proactively. Challenges faced included senior managers not taking the time to ensure meetings were managed productively and an initial unwillingness of meeting participants to make decisions. These authors note that “*institutionalizing the new principles and practices intended to nurture collective problem-solving and collective responsibility for service improvement [takes] time”* [92]. They did, however, find that intervening in the existing meeting structures created emergent and positive changes such as building supportive relationships across organisational silos, as well as improved collective decision making and sensemaking in management meetings. They argue, as we do here, that managers at the local level must be given the flexibility to experiment, whilst nurturing relational leadership skills and distributional leadership can improve the practice of decision making [92].

Finally, we argue that building the capacity of the ‘structures’ (e.g. meetings, organisational processes) that hold the district health system are critical for developing capacity and unleashing the tools, skills and infrastructure in the system at large. Structural capacity includes decision making fora where inter-sectoral discussions occur and corporate decisions are made, records are kept and individuals are called to account for non-performance [93]. We call for more research to build our understanding of the challenges and opportunities for building the capacity of managers from the bottom-up in the district health system.

## Limitations

Improving district management team functioning is part of the long chain of proximal and distal outcomes needed to improve the capacity of district management teams towards improved responsiveness, equity and health outcomes. This research only provides insight into one cog of this wheel – that is, the social processes of sensemaking and sensegiving and their interaction with the hardware of the system needed to motivate change. We were also only able to observe short term outputs. Additional longitudinal research is needed to understand how bottom-up innovations are institutionalised over the long term and the consequences for long term health goals. Finally, we did not reflect here on all the challenges faced by the new DM when introducing the changes; these will be considered in a subsequent cross case analysis.

## Conclusion

We argue that local managers are well placed to design CD innovations and must draw on tacit and experiential knowledge and system thinking capacities in thinking ‘bottom-up’. As their commitment and motivation are required to engage in CD processes, senior managers with power must draw on both their individual software competencies and the hardware resources of the system to influence motivation for capacity development. The act of managing is an everyday process, and we posit that CD can, thus, be conceived of as an everyday act of managing in routine structures while simultaneously building structural capacity in routine organisational processes. We recommend that further research is undertaken to understand bottom-up capacity development from a systems perspective, as well as CD interventions targeted at system ‘structures’ and organisational processes.

## Supplementary Information

**Additional file 1.** Initial programme theory for strengthening management capacity. File 1 shows a picture of the initial programme theory, it complements the programme theory narrative in the manuscript.

**Additional file 2.** Interview guide. File 2 is an interview guide that was used to elicit the initial programme theory and was used to gather follow up information in later interviews with managers.

**Additional file 3.** Interview Guide with NGO partners. File 3 is an interview guide that was developed to elicit NGO partner assumptions and experiences with the innovation.

**Additional file 4.** Summary of results. File 4 is a summary of the results section in table format. It complements the results section in the manuscript.

**Additional file 5.** The full range of prospective agenda items. File 5 is a full summary of all the new agenda items that could prospectively be discussed in the monthly management meetings. It complements the discussion on the new agenda in the manuscript.

## Data Availability

The datasets generated and/or analysed during the current study are not publicly available as we did not receive consent from participants to share the full transcripts.

## Abbreviations

DM: District Manager
DMT: District Management Team
DHS: District Health System
IM: Information Manager
HMIS: Health Management and Information Systems Policy
NHI: National Health Insurance

## Footnotes

1 In the larger project, we selected 3 of the 11 NHI pilot sites based on the following criteria: (1) a district that was actively receiving information from other levels of government and/or was implementing some of the innovations, (2) access to the site, meaning district managers were prepared to give us access to staff and (3) rural/urban mix to capture variation in experiences of implementation possibly linked to geography (f40 et al., 2019).

2 As part of another innovation in the district some hospital managers were being replaced in their current job due to new job requirements, they were not removed from the payroll or from the district.

3 These were not new formal posts on the organogram but rather a description of the duties they were expected to fulfil.
