# Supplementary material for: Bottom-up innovation for health management capacity development: a qualitative case study in a South African health district
Source: BMC Public Health. 2021 Mar 24;21:587. doi: 10.1186/s12889-021-10546-w (PMC7992952; doi:10.1186/s12889-021-10546-w)
Supplement: Supplementary file 1 — Additional file 1. Initial programme theory for strengthening management capacity. File 1 shows a picture of the initial programme theory, it complements the programme theory narrative in the manuscript. [file 12889_2021_10546_MOESM1_ESM.pptx]

## Slide 1
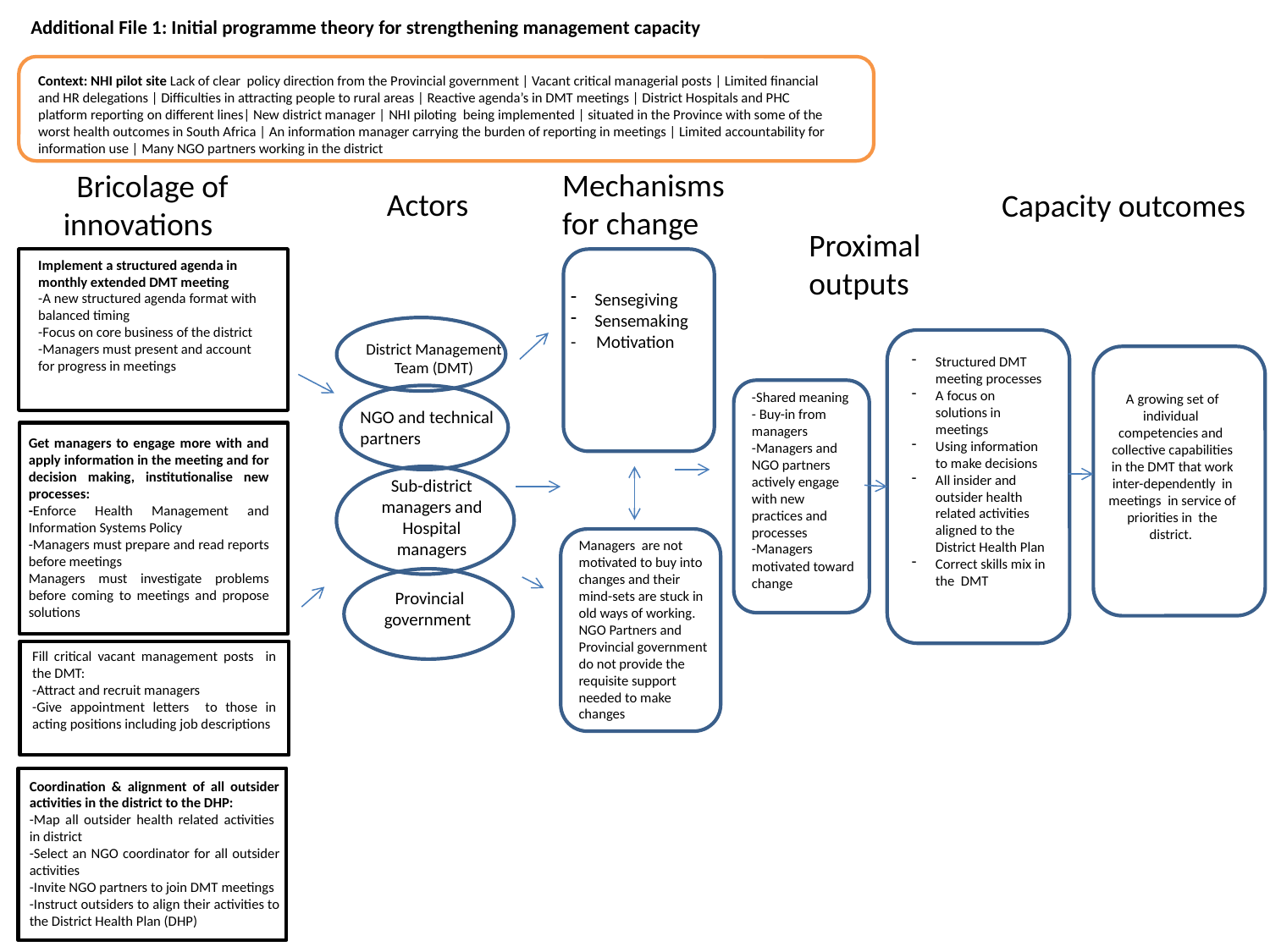

Additional File 1: Initial programme theory for strengthening management capacity
Context: NHI pilot site Lack of clear policy direction from the Provincial government | Vacant critical managerial posts | Limited financial and HR delegations | Difficulties in attracting people to rural areas | Reactive agenda’s in DMT meetings | District Hospitals and PHC platform reporting on different lines| New district manager | NHI piloting being implemented | situated in the Province with some of the worst health outcomes in South Africa | An information manager carrying the burden of reporting in meetings | Limited accountability for information use | Many NGO partners working in the district
Mechanisms for change
 Bricolage of innovations
Actors
Capacity outcomes
Proximal outputs
Implement a structured agenda in monthly extended DMT meeting
-A new structured agenda format with balanced timing
-Focus on core business of the district
-Managers must present and account
for progress in meetings
Sensegiving
Sensemaking
- Motivation
District Management Team (DMT)
Structured DMT meeting processes
A focus on solutions in meetings
Using information to make decisions
All insider and outsider health related activities aligned to the District Health Plan
Correct skills mix in the DMT
-Shared meaning
- Buy-in from managers
-Managers and NGO partners actively engage with new practices and processes
-Managers motivated toward change
A growing set of individual competencies and collective capabilities in the DMT that work inter-dependently in meetings in service of priorities in the district.
NGO and technical partners
Get managers to engage more with and apply information in the meeting and for decision making, institutionalise new processes:
-Enforce Health Management and Information Systems Policy
-Managers must prepare and read reports before meetings
Managers must investigate problems before coming to meetings and propose solutions
Sub-district managers and Hospital managers
Managers are not motivated to buy into changes and their mind-sets are stuck in old ways of working.
NGO Partners and Provincial government do not provide the requisite support needed to make changes
Provincial government
Fill critical vacant management posts in the DMT:
-Attract and recruit managers
-Give appointment letters to those in acting positions including job descriptions
Coordination & alignment of all outsider activities in the district to the DHP:
-Map all outsider health related activities in district
-Select an NGO coordinator for all outsider activities
-Invite NGO partners to join DMT meetings
-Instruct outsiders to align their activities to the District Health Plan (DHP)
