# Supplementary material for: Bottom-up innovation for health management capacity development: a qualitative case study in a South African health district
Source: BMC Public Health. 2021 Mar 24;21:587. doi: 10.1186/s12889-021-10546-w (PMC7992952; doi:10.1186/s12889-021-10546-w)
Supplement: Supplementary file 2 — Additional file 2. Interview guide. File 2 is an interview guide that was used to elicit the initial programme theory and was used to gather follow up information in later interviews with managers. [file 12889_2021_10546_MOESM2_ESM.docx]

**Additional File 2: Interview guide**

Key questions asked to elicit assumptions and activities related to management strengthening over time:

- What is your overall vision/goal?
- What are the key activities you will implement to achieve that vision/goal and who will be involved in implementing them and in taking action to achieve that vision/goal?
- What assumptions underpin the selection of these activities in relation to this vision/goal?
- What assumptions underpin the actors you will involve, or expect to play a role, and the expectations you have of their role in taking action towards the vision/goal?
- Who will be involved in implementing them and in taking action to achieve that vision/goal?

Other items to elicit assumptions and activities:

- What are the main types of changes that we want to support?’
- What are the development conditions that need to be in place for change even to be possible?’
- Identify the 3 most important relationships between these conditions?

Other factors to consider from the perspective of beneficiaries:

- How would they know about your programme?
- How would they understand it?
- Why should they participate?
