# Supplementary material for: Bottom-up innovation for health management capacity development: a qualitative case study in a South African health district
Source: BMC Public Health. 2021 Mar 24;21:587. doi: 10.1186/s12889-021-10546-w (PMC7992952; doi:10.1186/s12889-021-10546-w)
Supplement: Supplementary file 3 — Additional file 3. Interview Guide with NGO partners. File 3 is an interview guide that was developed to elicit NGO partner assumptions and experiences with the innovation. [file 12889_2021_10546_MOESM3_ESM.docx]

**Additional File 3 : Interview guide for NGO partners**

**General**

- Please state your organisation and your role in this organisation
- How long have you worked for this organisation in this role / and in this district
- Please describe the goal and objectives of your organisation in district X
- Are you aware that District X is an NHI pilot site / is this the reason your organisation works in District X

Prompts: (own internal organisation context; DMT number and competency of managers; support systems – use of information, environment – hierarchies; Province; National etc.)

- Could you reflect on your understanding and experience of the context within which you work in District X

**Coordination and collaboration**

- Please discuss your engagements with the district X management team
- Who do you work with and on what issues?
- What meeting spaces do you engage with members of the DMT
- Do you have the sense that you share a common vision for the district with the DMT?
- Are there any challenges or opportunities you would like to reflect on in this regard?

Note to participant: In 2014 the District manager tried to institutionalise mechanisms to strengthen coordination and collaboration between the DMT and the partners in the district; are you aware of any of these mechanisms and did you participate in them, if so how and why? Is there a functioning coordination mechanism in the district to ensure good coordination and collaboration in the district (reflect on pre and post 2014)

**Prompts**: invitations to DMT meetings; mapping all partner activities in the district, appointed a coordinator for all partners; joint planning of the District Health Plan including province.

- Do you share information with each other? What type, with whom and for what purpose?
- Do you share resources (e.g. monetary or skills etc.) What type, for whom and for what purpose?
- Do you partake in any joint action with the DMT for the benefit of the district? What type, with whom and for what purpose?
- Could you discuss any key challenges or opportunities these activities present? Please describe why and how these challenges present themselves.
- Has working with the DMT in any way affected your autonomy in the District?
- Do internal or external hierarchies come into play in your work with the district?
- Are you always able to reach task consensus? If yes, how?
- If you feel that in 2014 coordination and collaboration improved between the partners and the district could you please describe why you feel this way? What has changed and to what do you attribute these changes to?
- Are you now regularly (monthly) invited to participate in DMT meetings?
- Are you regularly invited to attend planning meetings jointly with the district? Do you collaborate on the joint development of business plans or strategies? If yes when and on what topics?
- Are the formal structures (routine meetings, institutions or bodies) that enable you to participate in the DMT activities?

**Institutionalization questions:**

- Is it now standard practice to participate in these activities? When and where?
- What spaces (geographic and temporal) are used to facilitate these activities?
- Are invitations sent out to participate routinely? Are the same actors always involved?
- Why do you participate in this activity, what motivates you to be a part of it?
- Are there sufficient resources / staff to carry out this activity? (Find out if there are any resources allocated for this, often is an indication of its importance).
- Has there been any communication strategy about this activity? How did you find out about it?
- Who sends out notifications? Who do you see as the lead person on this in the district?
- Are events / spaces of engagement well organized and facilitated in a structured manner?
- Do you a sense that in the current contextual environment that this activity is needed within the district context (district fit)?
- Do you have any comments on the target audience of this activity / and or the implementers of this activity
- Would you say these activities are now part of the normal day to day business of the district (sub district) or are there still some teething issues?
- What underlying mechanisms enabled the institutionalization of this activity?
