# Supplementary material for: Bottom-up innovation for health management capacity development: a qualitative case study in a South African health district
Source: BMC Public Health. 2021 Mar 24;21:587. doi: 10.1186/s12889-021-10546-w (PMC7992952; doi:10.1186/s12889-021-10546-w)
Supplement: Supplementary file 4 — Additional file 4. Summary of results. File 4 is a summary of the results section in table format. It complements the results section in the manuscript. [file 12889_2021_10546_MOESM4_ESM.docx]

**Additional File 4: Summary of results**

| **Initial context** | **The bricolage of innovations** | **Mechanisms (reasoning and resources) triggered in context.** | **Proximal output** | **Emergent outcomes (see Table 3 in text for description)** |
| --- | --- | --- | --- | --- |
| Historically under-resourced district, poor health outcomes relative to other districts in the country.    Critical vacancies in the management team and management meetings not functioning at optimal levels.  A need to implement multiple new interventions linked to NHI piloting.  A new district manager arrives from another Province in the district with years of experience in the public and private health system. | The initial process of designing the bottom up bricolage of innovations | **Resources of the new DM:**   - Positional authority of being the new DM (new DM in the context as trigger for change) - Networking capabilities   **Sensemaking by the DM drawing on:**   - Complex leadership skills - Systems thinking skills - Tacit knowledge and experiential knowledge from years of service in the health system - Hardware (rules and regulations) of the system - The capacity to engage in bricolage, the ability to pull together a range of innovations that work together in complementary ways - Focuses on doing more with what they have available, not limited by limited resources - Acknowledges that managers must make hard decisions | A set of innovations designed to improve the functioning of the DMT monthly management team meetings (structural capacity). | The individual competencies of the district manager contribute to the emerging capacity of the district management team. |
| -Meetings were not well structured, lots of complaining and time not spent on core business.  - Lack of preparation before meetings by sub DMS  - People falling asleep because of long presentations  - DM considers the district as an unstable system and says he must first institutionalise functional systems.  -This is a historically underperforming district.  -Lack of accountability | **Innovation 1a**  The introduction of a new agenda that focused on the core functions of the district, addressing the system building blocks (the agenda included ‘services’, ‘corporate governance’ and ‘quality’, with time allocated for each item) and the introduction of a routine procedure whereby managers have to produce reports covering core indicators for reading and distribution before the meeting. | **Primary resource: the new agenda (hardware)**  **Resources of the new DM:**   - Tacit knowledge and experiential knowledge from years of service in the health system to develop the new agenda - Positional authority to direct change - Systems thinking skills   **DM acts of sensegiving:**   - The new DM *translates* the need for a new agenda in familiar discourses of the public sector (need for a focus on core business, PHC and performance) - *Discipling the space* by allocating time for all important issues and directing who must present when and what must be prepared before the meeting - Further *disciplines the space* and *justifies change* by naming the rules of the ‘auditor-general’ to subjectively influence managers.   **Social processes of sensemaking by the DMT:**   - Proximity of managers to the ideas underpinning change generates motivation - Action provides more ingredients for sensemaking, as managers engage with the new practices they see more of its value and are motivated to engage further as they improve self-efficacy.   **Evolving context:**   - By 2015 the national government was also implementing broader processes for discussions on performance and performance monitoring | The structured agenda covering core business of the district was being routinely applied.  Managers had time allocated for giving input on their targets and indicators in meetings.  Reports were being generated by managers before meetings. | Improving and emerging capability to commit and engage in the DMT extended meetings. |
| Managers were not engaging well with information to support problem diagnosis and decision making  The information manager routinely provided the summary presentation of information in the meeting when discussing the performance of districts.  An over reliance on the information manager to deal with ‘numbers’ issues by managers in the district  Limited accountability for information use by other managers  A focus on problems and complaining in meetings, with limited focus on solutions. | **Innovation 1b**  An explicit effort to get DMT members to engage more with and apply information in meetings and for decision making by enforcing the HMIS policy.  Managers must prepare and read reports before meetings. And speak to sub district progress in meetings.  Managers must investigate sources and causes of problems before bringing them to the monthly meeting and be ready to discuss solutions. | **Resources of the DM:**   - Positional authority as the new DM to enforce new practices - Experiential knowledge of the health system   **DM acts of sensegiving:**   - Draws on the hardware resources of the bureaucracy (over-coding) in the form of the HMIS policy to enforce and *justify change* - *Over coding*, the inclusion of the use of data in senior manager performance contracts to reinforce meaning - Sets the scene by drawing on the motivation and knowledge of the information manager (symbolic of information) to further craft legitimacy and translate change - Visits managers with the information manager at their facilities to *justify the change* and ‘*setting the scene’* - Managers must produce and read reports before the meeting - *Disciplines the space* through the new agenda, timing and presenters - *Discipling the space* through distributed leadership*,* drawing on the IM is symbolic of the overall goal of information use.   **Social process of sensemaking by the DMT:**   - New resources such as 3g cards and laptops reinforced a focus on information use in the broader environment. - Some managers in the DMT had become permanent staff members in the period, this reinforces a sense of accountability. | Service delivery information increasingly being presented and discussed in the meeting to enable decision making and managers had to account for targets.  The information manager had a clear role to present information only.  The use of information was now part of each managers performance contract.  Managers increasingly bringing information from the ground to illustrate problems and discuss progress and learning. | Improving and emerging capability to commit and engage in the DMT extended meetings. |
| Some good historical relationships between NGO partners and the district already exist (a few difficult relationships also)  Many NGOs operating in the district, unclear whether they were working toward the district goals in the DHP  Unclear whether NGOs have a shared vision  As an NHI pilot site, more actors arriving in the district | **Innovation 1c**  -The routine procedure that NGO partners in the district would attend the extended management meeting in order to support coordination and accountability, as well as discuss their activities directly with the DM | **Resources of the DM**   - Positional authority of the DM to call actors together - Network power   **DM acts of sensegiving**   - *Disciplining the space*, calls NGO partners together to report to him on what they are doing to reinforce new expectations. - *Translating change*, gives voice to NGO partners to create shared meaning - *Sticks,* will report NGOs to funders who do not want to create shared vision - The new DM knows some of the NGO staff at a large NGO head office and negotiates for extra resources - *Discipling the space* through distributed leadership, the planning manager is called on to steward the NGOs   **Social process of sensemaking by the DMT:**   - Taps into intrinsic motivation of NGO partners who believe in serving the district and who have past working relationships [path dependency] - Shared sense of understanding in the DMT that the NGOS must work toward the goals in the district - The largest NGO has a specific mandate from the National government to be there and work with the district - In the context there are very specific projects (UNICEF) that reinforce working relationships between managers and NGOs in a good way. - The NGOs are also working with the DMT in developing annual district health plans. | By 2015 a growing number of NGOs were attending extended DMT meetings, presenting on progress  An increasing number of NGOs are now also part of developing the District Health Plan. | Improving and emerging capability to relate and to attract support |
| Critical vacancies in the management team and no additional funding to secure posts.    Hospital CEOs who had to leave their posts due to new job requirements were sent to work in the district office with no specific portfolio. | **Innovation 1d**  Finding a meaningful purpose for the ex-Hospital CEOs  Efforts to fill critical management vacancies in the DMT. | **Resources:**   - Positional authority as the DM to negotiate for posts and define roles   **DM acts of sensegiving**   - Justifies the need for a quality assurance manager by getting managers to experience the process of ranking to subjectively influence them. - Negotiates with the Provincial government - Provides the ex-Hospital CEOs with role descriptions is *symbolic* of their role in the team (Translation is not a neutral act, elements and symbols are chosen purposefully to establish shared meaning) | The DM fills a critical managerial post in the DMT (a quality assurance manager).  The Hospital CEOs have clear roles that are aligned to the needs in the District Management Team and feel they know what they need to do. | Improving and emerging capability to commit and engage in the DMT extended meetings. |
