# Supplementary material for: Bottom-up innovation for health management capacity development: a qualitative case study in a South African health district
Source: BMC Public Health. 2021 Mar 24;21:587. doi: 10.1186/s12889-021-10546-w (PMC7992952; doi:10.1186/s12889-021-10546-w)
Supplement: Supplementary file 5 — Additional file 5. The full range of prospective agenda items. File 5 is a full summary of all the new agenda items that could prospectively be discussed in the monthly management meetings. It complements the discussion on the new agenda in the manuscript. [file 12889_2021_10546_MOESM5_ESM.docx]

**Additional File 5: The full range of prospective agenda items**

**Priorities**

**Strategic objectives**

Womens Health

Services

Child Health

Community based services

Infectious diseases

Acute services

Ambulatory care

Managing the Burden of Disease

Source: Personnel communication with the District Manager

**Clinical Governance**

Norms & Standards

Maintenance and Laundry Management

Staff attitudes

Information management

Policy and Planning

Health Impact assessment

Professional support

Expenditure review (DHER)

Competency based teams

**Benchmark and performance indicators per domain**

Improving the quality of health services

Quality

Corporate Governance

Patient safety

Infection control

Cleanliness

Client experience

HRP & Equipment

Infrastructure management

Appropriate health technology and infrastructure

Strategy and Health support

Developing a capacitated workforce

Effective financial control

Personnel Targets

Establishment management

Audit compliance

Budget management
